# Self-Heating-Induced Deterioration of Electromechanical Performance in Polymer-Supported Metal Films for Flexible Electronics

**DOI:** 10.1038/s41598-017-12705-9

**Published:** 2017-10-02

**Authors:** Dong-Won Jang, Jeong-Hwan Lee, Ansoon Kim, Soon-Bok Lee, Seong-Gu Hong

**Affiliations:** 1 Reliability Assessment Center, Korea Institute of Machinery and Materials, Daejeon, 34103 Republic of Korea; 2 Division of Industrial Metrology, Korea Research Institute of Standards and Science, Daejeon, 34113 Republic of Korea; 3 Department of Nano Science, University of Science and Technology, Daejeon, 34113 Republic of Korea; 4 Department of Mechanical Engineering, Korea Advanced Institute of Science and Technology, Daejeon, 34141 Republic of Korea

## Abstract

The retention of electrical performance under the combined conditions of mechanical strain and an electrical current is essential for flexible electronics. Here, we report that even below the critical current density required for electromigration, the electrical current can significantly deteriorate the electromechanical performance of metal film/polymer substrate systems. This leads to a loss of stretchability, and this effect becomes more severe with increasing strain as well as increasing current. The local increase of electrical resistance in the metal film caused by damage, such as localized deformations, cracks, etc., locally raises the temperature of the test sample via Joule heating. This weakens the deformation resistance of the polymer substrate, accelerating the necking instability, and consequently leading to a rapid loss of electrical conductivity with strain. To minimize such a current-induced deterioration of the polymer-supported metal films, we develop and demonstrate the feasibility of two methods that enhance the deformation resistance of the polymer substrate at elevated temperatures: increasing the thickness of the polymer substrate, and utilizing a polymer substrate with a high glass transition temperature.

## Introduction

Flexible electronics is an emerging technology that has potential in a wide variety of applications, including flexible displays^1–4^, artificial skins^5^, stretchable circuits^6^, solar cells^7^, and wearable sensors^8^. In flexible electronics, most components lie on a compliant polymer substrate and are connected with each other using metallic interconnects. Thus, their functionality and usability rely entirely on the electromechanical performance of the metal film/polymer substrate couple under operational conditions. In this sense, sustaining a large deformation while maintaining a low and constant electrical resistance (i.e., achieving high stretchability) is considered an essential requirement for the implementation of flexible electronics. Considerable effort has been devoted in this regard to date^9–15^. It is noted, however, that flexible electronic devices are subjected to electrical currents as well as mechanical strain under working conditions. The electrical current can influence the electromechanical performance of the metal film/polymer substrate system in two ways: 1) the loss of conductivity of the metal film by electromigration^16–18^ and 2) the improvement of the metal film ductility by Joule heating^19,20^ or electroplasticity^21–23^. The current density, usually required in state-of-the art flexible electronic devices, is less than ~5 × 10^4^ A cm^−2^, somewhat below the critical value required for electromigration^24^; hence, this effect is expected to be negligible. In addition, considering the high melting temperatures of metal films (e.g., 961 °C for a silver (Ag) film^25^ the temperature rise caused by Joule heating appears not to have a significant effect on ductility enhancement. For these reasons, the effect of the electrical current on electromechanical behavior has been ignored in the design of metal film/polymer substrate systems. However, the trend toward smaller and more functional and powerful devices demands an increasing current density, together with a much higher stretchability. The high current density will raise the possibility of electromigration under operational conditions. It is known that large deformations in the metal film give rise to inhomogeneities (i.e., localized deformation, formation of cracks, etc.)^10,26,27^. These will lead to localized Joule heating, causing a temperature rise in both the metal film and polymer substrate. These points therefore suggest that the electrical current may play an important role in the electromechanical performance of future flexible electronic devices.

In this study, we investigated the electromechanical behavior of Ag film/polyethylene terephthalate (PET) substrate systems under electrical currents. Tensile tests were performed in combination with electrical resistance measurements, inside a scanning electron microscope (SEM) or under an infrared (IR) camera, while applying a constant electrical current. The current-induced deterioration of electromechanical performance was analyzed in a quantitative manner by defining the stretchability of the material system. An underlying mechanism of the deterioration was inferred, focusing on the Joule heating-induced acceleration of the necking instability in the PET substrate. We further suggested a viable solution to prevent (or reduce) the current-induced deterioration of electromechanical performance by using a polymer substrate with a high glass transition temperature, and demonstrated its feasibility with a polyimide (PI) substrate.

## Results and Discussion

### Electromechanical behavior and its variation under electrical currents

**Figure 1:**
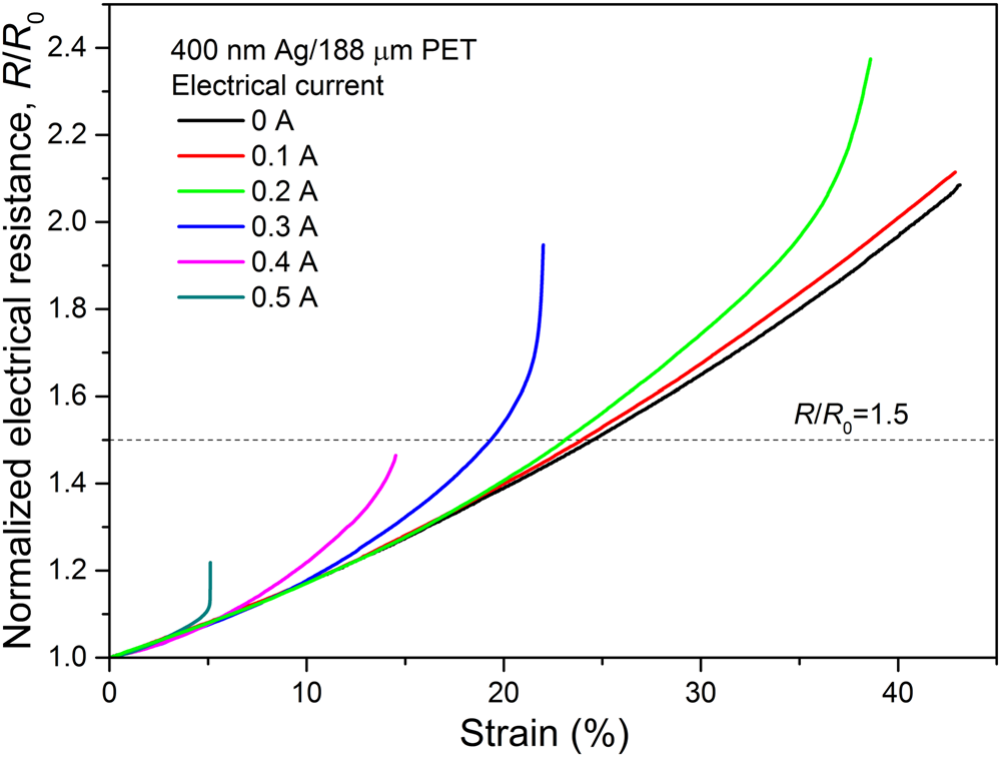
Electromechanical behavior (normalized electrical resistance−strain curve) of 400 nm Ag/188 μm PET and its variation under an electrical current; *R* and *R*_0_ are the electrical resistances at the strained and unstrained states, respectively.

Figure 1 shows the normalized electrical resistance−strain curves of 400 nm Ag/188 μm PET with and without an electrical current (*I*). The reproducibility was confirmed by employing each test condition more than three times; representative curves are presented. The electrical resistance generally increased with increasing strain, irrespective of the application of an electrical current. Notably, the rates of the resistance increase with strain were distinctly different. When no electrical current was applied, the electrical resistance gradually increased with strain up to *ε* > ~40%, indicating that no severe damage (e.g., cracks) developed in the Ag film (refer to Figure S1 in the Supplementary Information for the SEM micrographs showing the surface morphologies of this sample at six strain levels). However, the application of an electrical current caused deviating behavior. Once the divergence between the curves with and without an electrical current started to occur, the extent of deviation became more pronounced with further strain, accelerating the loss of electrical conductivity and consequently leading to an early failure of the sample. The onset strain at which the curves began to deviate decreased with increasing electrical current (*ε* = 19% for *I* = 0.1 A; *ε* = 17% for *I* = 0.2 A; *ε* = 10% for *I* = 0.3 A; *ε* = 6% for *I* = 0.4 A; and *ε* = 3% for *I* = 0.5 A), and the extent of the deviation became more severe with increasing electrical current. The current has a negligible effect on electromechanical behavior at the early stage of deformation, as shown by the matching curves in Fig. 1 in this region. It is clear, though, that its effect becomes important with increasing strain as well as increasing electrical current, deteriorating the electromechanical performance of the Ag film/PET substrate. The data for the 100 nm Ag/188 μm PET and 200 nm Ag/188 μm PET systems are provided in Figure S2, and consistent results with 400 nm Ag/188 μm PET were also confirmed.

**Figure 2:**
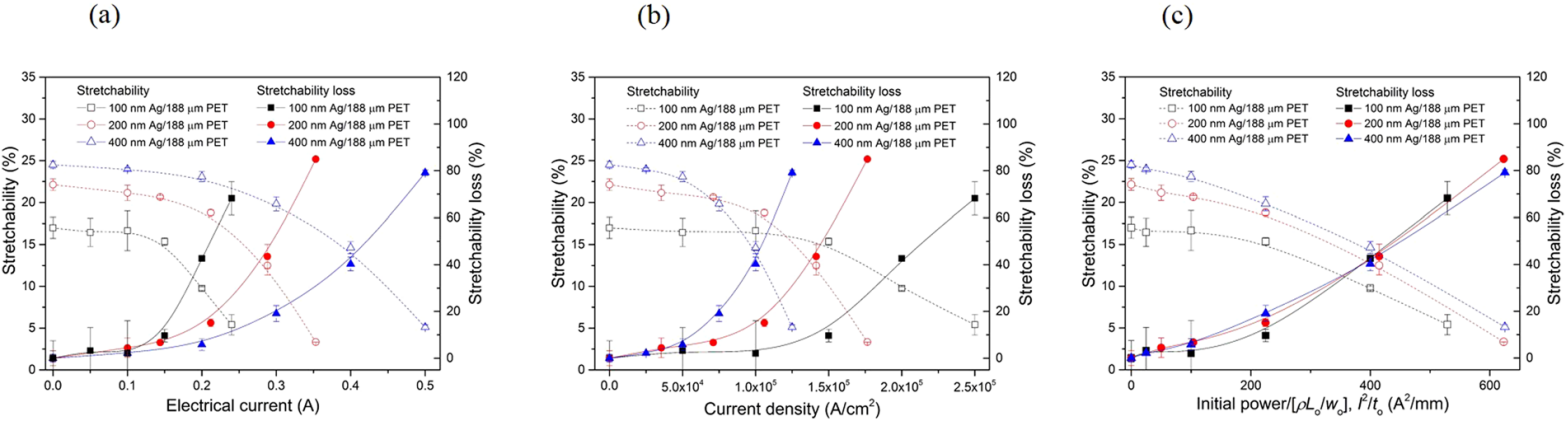
Stretchability and its loss for Ag film/PET substrate samples as a function of (**a**) electrical current, (**b**) current density, and (**c**) electrical power.

To quantitatively analyze the current-induced deterioration of electromechanical performance, we defined the stretchability of the Ag film/PET substrate as the strain value at which the normalized electrical resistance, *R*/*R*_0_, reached 1.5, i.e., a 50% increase relative to the initial resistance value. As shown in Fig. 1 and Figure S2, samples subjected to a high electrical current failed mechanically before reaching a value of *R*/*R*_0_ = 1.5. In these cases, the stretchability was defined as the mechanical failure strain of the sample. Figure 2a shows the variation of stretchability with electrical current. For all three systems with different Ag film thicknesses, the stretchability slowly decreased with increasing strain at low electrical currents, but a steep reduction was observed beyond a certain strain. The higher stretchability of the thicker Ag film without an applied electrical current is consistent with previous studies^28,29^. The electrical current at which a sharp decline in stretchability occurred was proportional to the Ag film thickness, and the rate of stretchability degradation was more severe in the 100 nm Ag/188 μm PET system than in the 200 nm Ag/188 μm PET and 400 nm Ag/188 μm PET systems. To systematically analyze the effect of the Ag film thickness on the current-induced deterioration of stretchability, the “stretchability loss” parameter was defined as follows:

1 $${\rm{stretchability}}\,{\rm{loss}}=\frac{{{\rm{stretchability}}|}_{I=0{\rm{A}}}-{\text{stretchability}|}_{I}}{{{\rm{stretchability}}|}_{I=0{\rm{A}}}},$$

where ${\mathrm{stretchability}|}_{I=0{\rm{A}}}$ and ${\mathrm{stretchability}|}_{I}$ are the stretchability values without and with an electrical current, respectively. The stretchability loss measures the current-induced reduction of stretchability with respect to the reference value without an electrical current. As shown in Fig. 2a, the effect of the Ag film thickness on stretchability loss was negligible at low electrical currents up to ~0.15 A, but it was clearly evident beyond this value. The thinner Ag film showed a pronounced stretchability loss and an increased degradation rate with increasing electrical current. This behavior suggests that the current-induced deterioration of electromechanical performance was accelerated in the thinner Ag film. However, when the stretchability data are analyzed in terms of the current density (*J*), the opposite results are obtained (Fig. 2b). The current density at which the stretchability started to rapidly decrease was inversely proportional to the Ag film thickness. Additionally, the rate of the stretchability decrease (or the stretchability loss increase) with electrical current was larger in the thicker Ag film. These trends indicate that the current-induced deterioration of electromechanical performance was accelerated in the thicker Ag film. These contrary results appear to arise from the fact that neither the electrical current nor the current density is a suitable parameter for explaining the current-induced deterioration of electromechanical performance. The electrical current does not account for the effect of sample size, particularly the cross-sectional area of the Ag film. The current density is considered a key parameter influencing the electrical performance deterioration of metal film/polymer substrate systems via electromigration^16–18^. Nevertheless, the SEM images showing the surface morphologies of the tested samples revealed that electromigration does not play a significant role in the present study; a detailed discussion in this regard will be presented later. Another factor that can be considered is the current-induced Joule heating effect. As the temperature rise is proportional to the electrical power applied^30–33^, we introduced a parameter associated with the electrical power, *I* ^2^/*t*_o_, which is equal to the initial power divided by the factor *ρL*_o_/*w*_o_. *ρ* is the electrical resistivity, and *L*_o_, *w*_o_, and *t*_o_ are the length, width, and thickness of the Ag film, respectively, at the unstrained state. Note that *ρ* is a constant value (~2 μΩ·cm in the film thickness range of ~80 to 1,000 nm^34,35^ *L*_o_ and *w*_o_ are fixed for all samples, and only *t*_o_ varies, indicating that the initial power applied to the sample is directly proportional to *I* ^2^/*t*_o_. The analyzed results are presented in Fig. 2c. Similar to the results obtained from the electrical current and current density measurements, stretchability loss occurred with increasing initial power (i.e., *I* ^2^/*t*_o_), but it occurred gradually and there was no drastic transition from the slow to steep increase. More importantly, all the data for the three different Ag film thicknesses resulted in a single stretchability loss−*I* ^2^/*t*_o_ curve, indicating that the Ag film thickness does not affect the current-induced deterioration of electromechanical performance. These findings indirectly support that the deterioration of the Ag film/PET substrate system observed in the present study arises from the current-induced Joule heating that leads to a temperature rise in both the film and substrate. The stretchability loss averaged over the three Ag film thicknesses was ~3% at *I* ^2^/*t*_o_ = 25 A^2^ mm^−1^ (corresponding to *J* = 2.5 × 10^4^ A cm^−2^ for 400 nm Ag/188 μm PET) and increased to ~6% at *I* ^2^/*t*_o_ = 100 A^2^ mm^−1^ (*J* = 5 × 10^4^ A cm^−2^). It was ~15% at *I* ^2^/*t*_o_ = 225 A^2^ mm^−1^ (*J* = 7.5 × 10^4^ A cm^−2^) and ~40% at *I* ^2^/*t*_o_ = 400 A^2^ mm^−1^ (*J* = 10^5^ A cm^−2^). The relationships between *I*, *J*, and *I* ^2^/*t*_o_ for the samples are given in Table 1. The current density used in state-of-the art flexible electronic devices is in the range of <5 × 10^4^ A cm^−2^ and electromigration requires the sustained application of a high current density of >10^5^ A cm^−2^ (note that the duration of each test in the present study was shorter than 12 min)^24^. Considering this, our results clearly show that, even below the critical current density required for electromigration, electrical current can induce the deterioration of electromechanical performance. This effect will eventually impair (or stop) device performance and should thus be carefully considered in the design of metal film/polymer substrate systems used in flexible electronic devices.

**Table 1:** The relationships between *I*, *J*, and *I* ^2^/*t*_o_ (=initial power/[*ρL*_o_/*w*_o_]) in the samples.

*I* ^2^/*t*_o_ (A^2^/mm)	100 nm Ag		200 nm Ag		400 nm Ag	
	*I* (A)	*J* (A/cm^2^)	*I* (A)	*J* (A/cm^2^)	*I* (A)	*J* (A/cm^2^)
25	0.05	5 × 10^4^	0.07	3.55 × 10^4^	0.1	2.5 × 10^4^
100	0.1	10^5^	0.14	7.1 × 10^4^	0.2	5 × 10^4^
225	0.15	1.5 × 10^5^	0.21	1.06 × 10^5^	0.3	7.5 × 10^4^
400	0.2	2 × 10^5^	0.28	1.42 × 10^5^	0.4	10^5^
625	0.25	2.5 × 10^5^	0.35	1.77 × 10^5^	0.5	1.25 × 10^5^

### A mechanism for the current-induced deterioration of electromechanical performance

**Figure 3:**
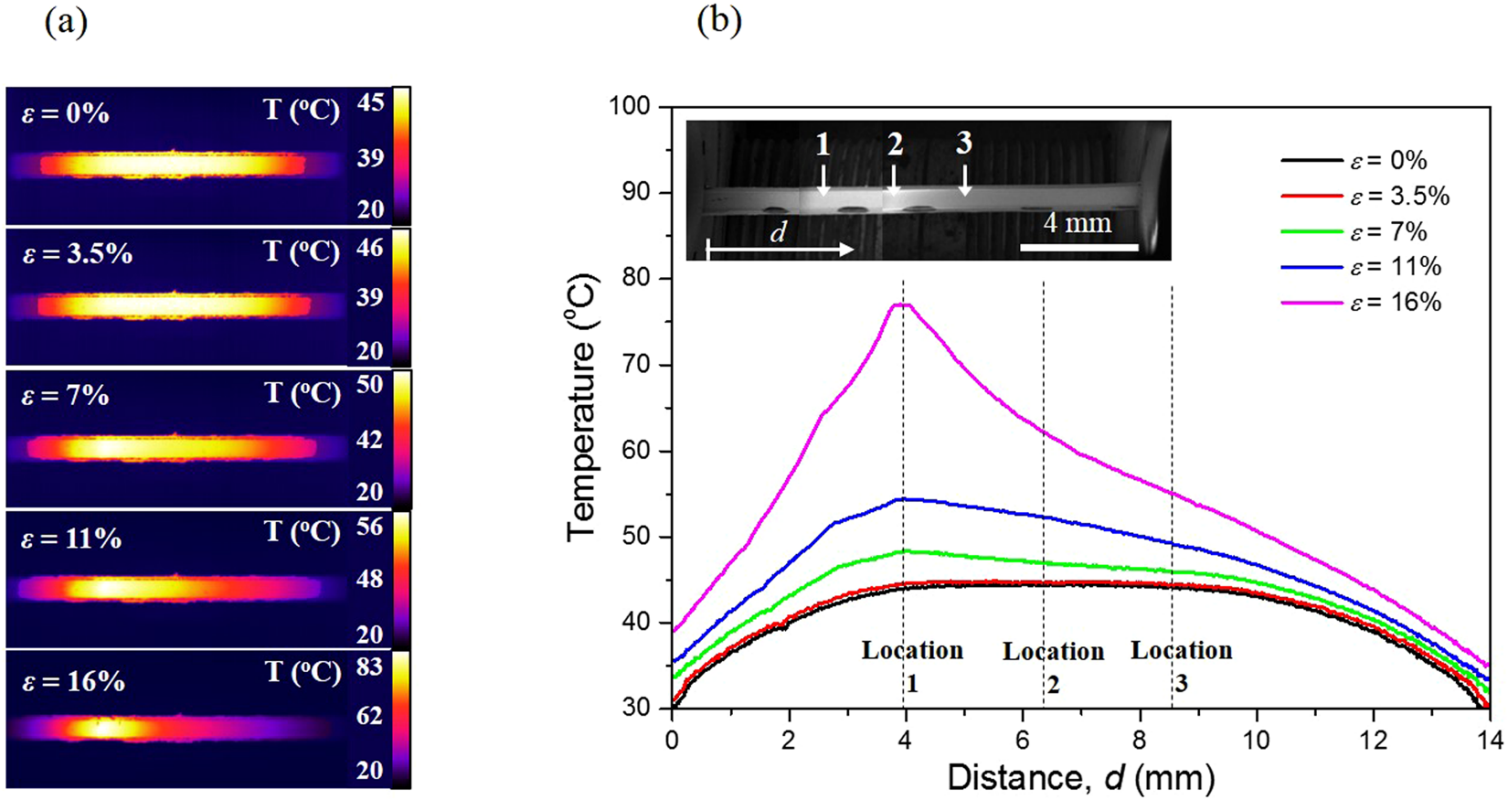
Temperature distribution of 400 nm Ag/188 μm PET during the tensile test with an electrical current of 0.3 A. (**a**) IR image and (**b**) the temperature distribution evolution with strain.

**Figure 4:**
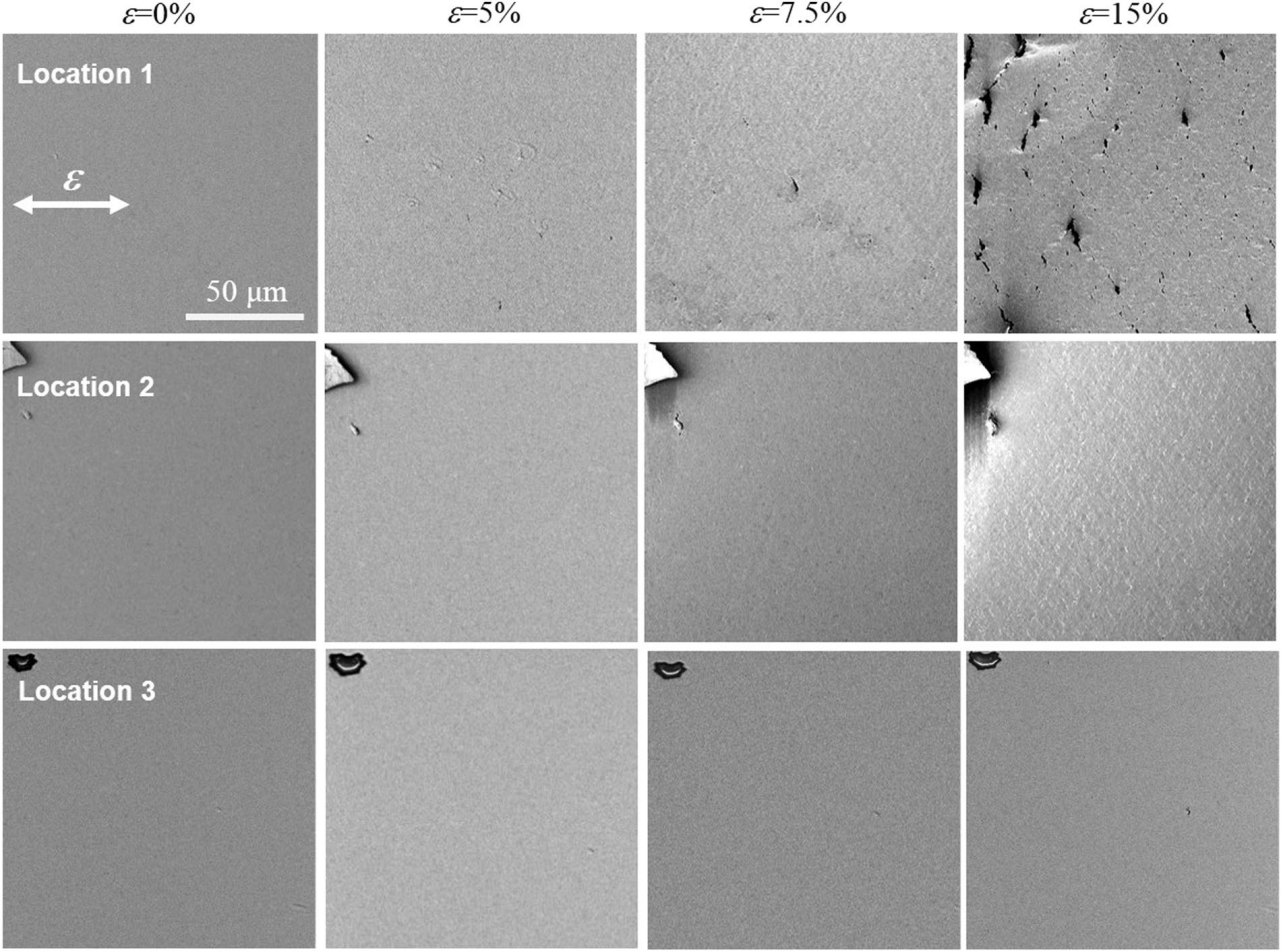
*In situ* SEM observations of three locations on 400 nm Ag/188 μm PET, indicated by 1, 2, and 3 in the inset of Fig. 3b, during the tensile test with an electrical current of 0.3 A.

As described earlier, the temperature rise in the samples caused by the current-induced Joule heating appears to be responsible for the deterioration of electromechanical performance. To test this hypothesis and understand the underlying mechanism, we measured the temperature distribution over the sample surface with an IR camera and observed the variation in surface morphology of the tested sample with strain using a SEM. The results for 400 nm Ag/188 μm PET under an electrical current of 0.3 A are presented in Figs 3 and 4. A uniform temperature existed at the center of the sample at low strains of <7%, which spanned ~7 mm or almost 50% of the sample gauge length (Fig. 3b); note that heat transfer from the sample reduced the temperature near the grips. However, a local temperature rise started to occur at *ε* = 7%, with the temperatures at locations 1 and 3 reaching 48 °C and 46 °C, respectively. This nonuniform temperature distribution became much more pronounced with further strain: the temperatures were 54 °C and 49 °C at locations 1 and 3 for *ε* = 11%, and increased to 77 °C and 55 °C at *ε* = 16%. According to the SEM observations (Fig. 4), there were no marked changes in surface morphology up to *ε* = 5%, but small cracks started to form near location 1 for *ε* = 7.5%. The formation of these small cracks will have induced a loss of electrical conductivity at location 1, thereby increasing the electrical resistance and leading to localized Joule heating. This interpretation provides a rationale for the local temperature rise at *ε* = 7%, shown in Fig. 3, and further suggests that the localized cracking in the Ag film is closely associated with the nonuniform temperature distribution. The localized cracking process proceeded further with strain, which caused severe damage around location 1 and enhanced the local rise of temperature at this site, eventually accelerating the mechanical failure of the sample there (refer to the SEM micrographs at *ε* = 15% in Fig. 4). It is noted that the surface morphologies at locations 2 and 3 remained almost unchanged even at *ε* = 15%, which supports the observation that the cracking was localized near location 1. Such a current-induced acceleration of the damage process in the sample was also reflected in the electromechanical behavior. As shown in Fig. 1, the electrical resistance−strain curve of *I* = 0.3 A started to deviate from that collected without an electrical current at ε = ~10%. According to previous studies, oxidation^36,37^ or electromigration^16,17^ in the Ag film could be responsible for the loss of electrical conductivity (i.e., the increase of resistance). However, the SEM micrographs and scanning X-ray photoelectron spectroscopy analysis of the tested sample surface, shown in Figs 4 and S3, revealed no evidence of oxides or electromigration, the latter of which would be indicated by the accumulation of material (hillocks) at the anode and depletion (voids) at the cathode^38,39^. We confirmed this by measuring the variation in electrical resistance of 400 nm Ag/38 μm PET and 400 nm Ag/188 μm PET with time under an electrical current of 0.3 A in the unstrained condition. As shown in Figure S4, the electrical resistance and temperature in the samples remained almost constant for ~30 min. This indicates that the electrical current itself does not cause any damage. Considering that the time taken for each test was less than ~12 min, we can conclude that the effects of oxidation and electromigration are negligible in the present study.

As for the crack generation mechanism, we have reported this in our previous study where the electromechanical characteristics of the identical material systems (i.e., Ag/PET and Ag/PI) were studied under no electrical current^29^. According to the results, the tractions exerted by the Ag film on the interface arise in the vicinity of imperfections (e.g., any defects or discontinuities) and this causes local delamination of the Ag film from the substrate. Once delaminated, the Ag film becomes locally freestanding, allowing strain localization (i.e., local necking) and eventually leading to crack formation. The two processes, delamination and strain localization, promote each other and coevolve, ultimately leading to Ag film failure^9,12,40^. It is noted that as described earlier, the electrical current does not have a meaningful effect at the early stage of tensile deformation, indicating that the crack generation mechanism, suggested for no electrical current condition, is still valid under an electrical current condition; the effect of electrical current becomes significant after the Ag film is damaged.

**Figure 5:**
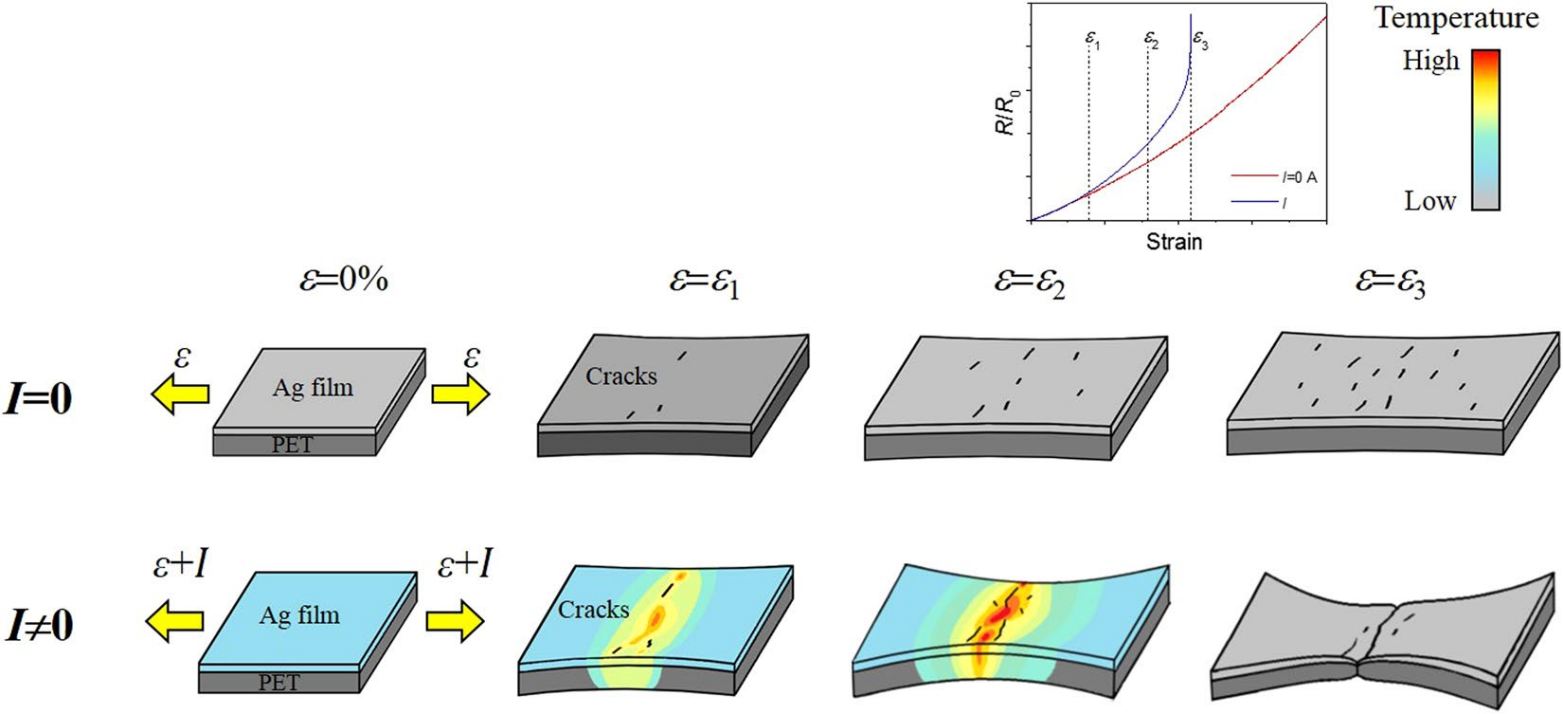
Schematic illustrating the fracture mechanism of the Ag film/PET substrate during the tensile test with or without an electrical current.

Based on these findings, we can speculate on the mechanism responsible for the current-induced deterioration of electromechanical performance in the Ag film/PET substrate system. At low strains, before any notable localized damage (localized deformations, cracks, etc.) develops in the sample, the deformation and hence the Joule heating occurs uniformly over the sample. With increasing strain, however, the deformation or crack formation is local to certain regions of the sample. This gives rise to a local increase of electrical resistance, thereby inducing Joule heating that locally raises the temperature both in the Ag film and PET substrate. Considering the high melting temperature of the Ag film (961 °C^25^), the temperature rise of <100 °C observed in the present study does not appear to have a significant effect on the ductility enhancement of the Ag film. It will, however, influence the deformation behavior of the PET substrate because of its low glass transition temperature (*T*_g_ = ~75 °C^41^). PET is therefore capable of facilitating deformation in the temperature range of the present study. The temperature dependence of the PET deformation behavior, obtained by conducting tensile tests inside a thermal chamber, is presented in Figure S5. The stress required to accommodate the strain was reduced by ~17% at 50 °C and ~25% at 70 °C in the strain range of 3–7%, indicating that PET deformed readily with increasing temperature. In the Ag film/PET substrate systems used here, the PET substrate was at least ~500 times thicker than the Ag film (470 times thicker in 400 nm Ag/188 μm PET and 1,880 times thicker in 100 nm Ag/188 μm PET). Therefore, the overall deformation behavior of each system was dominated by that of the PET substrate itself. The local rise of temperature will facilitate the deformation of the PET substrate at this location, inducing a localized deformation (i.e., necking instability), and this will accelerate cracking in the Ag film, consequently leading to a rapid loss of electrical conductivity with strain. A schematic illustrating the fracture mechanism of the Ag film/PET substrate during tensile tests performed with or without an electrical current is presented in Fig. 5.

### Strategy to improve the current-induced deterioration of electromechanical performance

It is known that a metal film on a polymer substrate can sustain a much larger deformation compared to its freestanding counterpart because of the suppression of the necking instability. The superior deformation capability of the polymer substrate suppresses the occurrence of localized deformation in the metal film, retarding the onset of necking, when the adhesion between the metal film and polymer substrate is strong enough to prevent delamination^10,40,42,43^. However, our results showed that such a beneficial effect is impaired under an electrical current due to the local Joule heating in the polymer substrate, which accelerates its necking instability and eventually leads to the reduction of stretchability. Considering the operational conditions of flexible electronic devices and the demand for increasing current densities together with a much higher stretchability in future devices, this current-induced deterioration of electromechanical performance is a critical issue that should be resolved for their commercialization.

**Figure 6:**
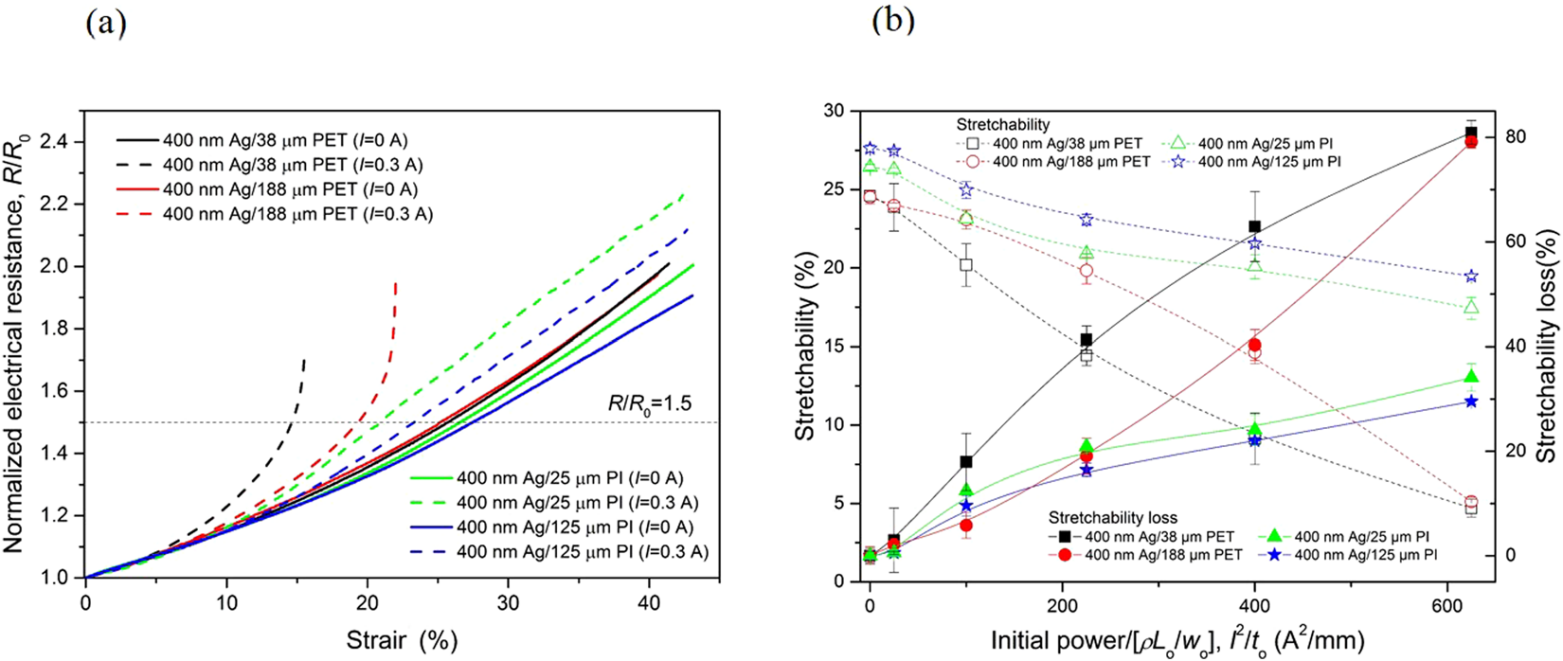
Improved electromechanical performance of the Ag film/PI substrate (or Ag film/thick PET substrate) systems under an electrical current. (**a**) Comparison of 400 nm Ag/PI substrates and 400 nm Ag/PET substrates under an electrical current of 0.3 A. (**b**) Stretchability and its loss as a function of electrical power for 400 nm Ag/PET substrates and 400 nm Ag/PI substrates.

According to our results, as the local heating and weakening of the polymer substrate is a primary cause of the current-induced deterioration of electromechanical performance, we can expect to improve the electromechanical performance of the polymer-supported metal film system under an electrical current by enhancing the deformation resistance of the polymer substrate at elevated temperatures. Two methods can be followed for achieving this purpose. One is to increase the thickness of the polymer substrate and the other is to utilize a polymer substrate with a high glass transition temperature. To demonstrate these methods, we varied the substrate thickness and material: 400 nm Ag/38 μm PET, 400 nm Ag/25 μm PI, and 400 nm Ag/125 μm PI samples were additionally fabricated and tested. Figure 6 compares the test results. Regarding substrate thickness, as shown in Fig. 6a, the electromechanical behavior of the 400 nm Ag/PET or 400 nm Ag/PI samples was almost identical irrespective of the substrate thickness when the electrical current was not applied. With the application of an electrical current, though, the deterioration of electromechanical performance (i.e., the increase of electrical resistance) with strain was accelerated for both the thin PET and PI substrates. The onset strain at which the curve of *I* = 0.3 A started to deviate from that of *I* = 0 A was *ε* = 2.9% for 400 nm Ag/38 μm PET; *ε* = 6.5% for 400 nm Ag/188 μm PET; *ε* = 8.0% for 400 nm Ag/25 μm PI; and *ε* = 9.5% for 400 nm Ag/125 μm PI. The stretchability (or stretchability loss) data, shown in Fig. 6b, also confirmed that the current-induced deterioration became more severe with a thinner substrate; the thick PET or PI substrates always possessed a higher stretchability (or a lower stretchability loss) than the thin versions in the electrical power range of *I* ^2^/*t*_o_ = 25 A^2^ mm^−1^ (*J* = 2.5 × 10^4^ A cm^−2^) to *I* ^2^/*t*_o_ = 625 A^2^ mm^−1^ (*J* = 1.25 × 10^5^ A cm^−2^). The underlying mechanism of the improved current-induced deterioration with increasing substrate thickness can be understood by considering that the higher heat capacity of the thick substrate mitigates the temperature rise of sample during the tensile test. This leads to a high deformation resistance, which consequently suppresses the necking instability in the thick substrate. It is noted that the effect of substrate thickness on the current-induced deterioration was noticeable with the Ag film/PET substrate samples, while it was minor in the Ag film/PI substrate samples. As for the effect of substrate material, the Ag films on the PI substrates with a high *T*_g_ of ~400 °C^41^ showed excellent electromechanical characteristics under electrical currents, compared to the Ag films on the PET substrates with a low *T*_g_ of ~70 °C^41^ (Fig. 6). Note that there was no marked difference in the electromechanical characteristics between the Ag films on PET and PI substrates without the application of an electrical current: the stretchability was ~25% for both 400 nm Ag/38 μm PET and 400 nm Ag/188 μm PET; 26% for 400 nm Ag/25 μm PI; and 27% for 400 nm Ag/125 μm PI. As explained earlier, the onset strain for the 400 nm Ag/25 μm PI system at which the *I* = 0.3 A and *I* = 0 A curves started to deviate was 8.0%, even higher than that of 400 nm Ag/188 μm PET (6.5%). The curves of 400 nm Ag/25 μm PI and 400 nm Ag/125 μm PI with *I* = 0.3 A did not significantly deviate from the curves of *I* = 0 A (Fig. 6a). Such an improved electromechanical performance with the Ag film/PI substrate system was reflected in the stretchability (or stretchability loss) data. As shown in Fig. 6b, the stretchability of the Ag film/PET substrate samples steeply reduced with increasing electrical power, but for the Ag film/PI substrate samples, the reduction of stretchability with electrical power was remarkably inhibited. The stretchability loss of the 400 nm Ag/38 μm PET was 3% at *I* ^2^/*t*_o_ = 25 A^2^ mm^−1^ (*J* = 2.5 × 10^4^ A cm^−2^) and increased to 81% at *I* ^2^/*t*_o_ = 625 A^2^ mm^−1^ (*J* = 1.25 × 10^5^ A cm^−2^), while that of 400 nm Ag/25 μm PI was 0.6% at *I* ^2^/*t*_o_ = 25 A^2^ mm^−1^ and 34% at *I* ^2^/*t*_o_ = 625 A^2^ mm^−1^. These results clearly demonstrate that the two suggested methods are effective for reducing the current-induced deterioration of electromechanical performance in the polymer-supported metal films. It is noteworthy that utilizing a polymer substrate with a high glass transition temperature is a more effective means of enhancing the deformation resistance at elevated temperatures, and increasing the thickness of polymer substrate can be thought of as a means of assistance. In addition, these findings again confirm that the mechanism of the current-induced deterioration suggested in the present study is reasonable.

At present, several research efforts are focusing on improving the stretchability of polymer/conductive material composite systems, which is essential for commercializing flexible electronics technologies. Promising methods include the geometrical manipulation of conductive materials (e.g., creating serpentine^44–52^, non-planar buckling^15,53–56^ or metal mesh structures^57–61^) and using nanowire networks^62–68^. Such methods are based on increasing the heterogeneity of the conductive material. However, as this raises the probability of local increases in electrical resistance, our results suggest that the reported improvements of stretchability will not be retained under the operational conditions of flexible electronic devices. The combined conditions of mechanical deformation and electrical currents will promote the local rise of temperature in conductive materials with increased heterogeneity, accelerating the necking instability and consequently leading to the reduction of stretchability. A thorough examination considering the working conditions of flexible electronic devices is therefore required to demonstrate the feasibility of the developed methods. It is further suggested that the two methods developed here to enhance the deformation resistance of polymer-supported metal films at elevated temperatures can be effective against this unwanted current-induced deterioration of electromechanical performance in polymer-supported conductive materials for flexible electronics.

## Conclusions

The electromechanical characteristics of polymer-supported metal films for flexible electronics were investigated under their operational conditions, i.e., a combination of mechanical deformation and electrical currents, by conducting electromechanical tensile tests inside a SEM or under an IR camera, while applying a constant electrical current. Our results showed that electrical current alone did not cause any noticeable loss of electrical performance. However, when combined with mechanical strain, it caused a remarkable loss of conductivity even below the critical value of current density required for electromigration. This current-induced deterioration of electromechanical performance became more severe with increasing electrical current as well as increasing strain. The *in situ* measurements of the surface morphology and temperature distribution over the samples during testing unveiled an underlying mechanism of the current-induced deterioration of electromechanical performance. The damage-induced local increase in the electrical resistance of the metal film, caused by localized deformations, cracks, etc., locally raised the temperature of the sample via Joule heating. This weakened the deformation resistance of the polymer substrate, accelerating the necking instability, and consequently leading to a rapid loss of electrical conductivity with increasing strain. To lessen such a current-induced deterioration of electromechanical performance in the polymer-supported metal films, we demonstrated the feasibility of two methods to enhance the deformation resistance of the polymer substrate at elevated temperatures: increasing the thickness of the polymer substrate and utilizing a polymer substrate with a high glass transition temperature. Our results further suggest that the current-induced deterioration of electromechanical performance would be a critical issue in the methods previously developed to improve the stretchability of various polymer-supported conductive materials for flexible electronics (e.g., creating serpentine, non-planar buckling or metal mesh structures, using nanowire networks, etc.) because they are mostly based on increasing the heterogeneity in the conductive material. The methods developed in the present study can be an effective means to resolve this issue.

## Methods

### Preparation of Ag film/PET substrate and Ag film/PI substrate samples

The polymer substrates used were PET films with thicknesses of 38 and 188 μm (Toray XG532, Toray Industries, Japan) and PI films with thicknesses of 25 and 125 μm (Dupont Kapton HN, DuPont, USA). The PET films were primer-coated on one side to improve their adhesion to the Ag film, while a primer coating was not needed on the PI films because of their good adhesion. These PET and PI substrates were patterned into 28 × 1 mm rectangles using a cutting plotter (Graphtec FC8000, Graphtec America, USA). Ag films with different thicknesses were deposited on the patterned PET and PI substrates using an electron-beam evaporator (KVET-C500200, Korea Vacuum Tech., South Korea) at a rate of 0.15 nm s^−1^. Ag films with thicknesses of 100, 200, and 400 nm Ag were deposited on 188-μm-thick PET substrates, and 400-nm-thick Ag films were deposited on the 38-μm-thick PET and 25- and 125-μm-thick PI substrates. The electrical resistance (*R*_0_ in Figs 1 and 6a), measured over a length of 14 mm and a width of 1 mm (i.e., the gauge section of the test sample), was 1.02 ± 0.13 Ω for the 100 nm Ag film, 2.56 ± 0.16 Ω for the 200 nm Ag film, and 6.10 ± 0.25 Ω for the 400 nm Ag film.

### Electromechanical tensile tests inside a SEM or under an IR camera

The gauge length of the above rectangular (28 × 1 mm) test samples excluding the two gripping parts was 14 mm. The electromechanical tensile tests were conducted using a commercial micro-tensile tester (Microtest, Deben UK Ltd., UK) in combination with simultaneous electrical resistance measurements. The strain developed in the sample was calculated by measuring the grip-to-grip distance whilst the strain rate was fixed at 6 × 10^−4^ s^−1^. A DC power supply (Keithley 2000, Keithley Instruments, USA) was used to apply a constant electrical current to the sample in the range of 0–0.5 A. To measure the variation of electrical resistance in the sample during the test, a voltage signal was collected with a digital multimeter (Keithley 2400 Multimeter, Keithley Instruments, USA) and was divided by the applied electrical current; two electrodes were located at the gripping parts, giving the 14 mm gauge length.

To observe the evolution of the surface morphology (i.e., damage) of the sample during the electromechanical tensile test, the test was conducted inside a SEM (Quanta FEG 650, FEI, USA). The instrument was operated at an accelerating voltage of 10 kV, at room temperature under high vacuum conditions of <5 × 10^−5^ mbar; the micro-tensile tester was installed in the sample holder of the SEM. In addition, the evolution of the temperature distribution over the sample surface during the test was measured using an IR camera (FLIR × 6580sc, FLIR Systems, Inc., USA). The surface of each sample was coated with a black dye to enhance the temperature measurements and a 5× magnification lens was used for obtaining the IR images. The temperature calibration of the IR camera system was performed by measuring the temperature of the sample with a K-type thermocouple and data acquisition unit (Keysight 34970 A, Keysight Technologies, USA) attached to the bottom of the polymer substrate; as the electrical current flowed in the Ag film during the test, it was impossible to attach the thermocouple directly to the Ag film. According to finite element analysis, the temperature difference between the top (Ag film) and bottom (polymer substrate) surfaces was negligible because of the thinness of the polymer substrate; for the 400 nm Ag/188 μm PET sample, the difference was less than 1 °C at a Ag film temperature of ~95 °C (refer to Figure S6). The schematic of the electromechanical tensile testing system operating inside the microscope or under the IR camera is presented in Figure S7.

### Data Availability

All data generated or analyzed during this study are included in this published article (and its Supplementary Information files.

## Electronic supplementary material

Supplementary Information

## References

[1] Chen Y (2003). Electronic paper: Flexible active-matrix electronic ink display. Nature.

[2] Gelinck G (2004). Flexible active-matrix displays and shift registers based on solution-processed organic transistors. Nature Materials.

[3] Kim S (2011). Flexible displays: low-power flexible organic light-emitting diode display device (Adv. Mater. 31/2011). Advanced Materials.

[4] Yoon B (2011). Inkjet printing of conjugated polymer precursors on paper substrates for colorimetric sensing and flexible electrothermochromic display. Advanced Materials.

[5] Ponce Wong R, Posner J, Santos V (2012). Flexible microfluidic normal force sensor skin for tactile feedback. Sensors and Actuators A: Physical.

[6] Kim D (2008). Stretchable and foldable silicon integrated circuits. Science.

[7] Docampo, P., Ball, J., Darwich, M., Eperon, G. & Snaith, H. Efficient organometal trihalide perovskite planar-heterojunction solar cells on flexible polymer substrates. *Nature Communications* 4, (2013). 10.1038/ncomms3761 24217714

[8] Kim D (2011). Epidermal electronics. Science.

[9] Xiang Y, Li T, Suo Z, Vlassak J (2005). High ductility of a metal film adherent on a polymer substrate. Applied Physics Letters.

[10] Li T (2005). Delocalizing strain in a thin metal film on a polymer substrate. Mechanics of Materials.

[11] Lacour S, Chan D, Wagner S, Li T, Suo Z (2006). Mechanisms of reversible stretchability of thin metal films on elastomeric substrates. Applied Physics Letters.

[12] Li T, Huang Z, Suo Z, Lacour S, Wagner S (2004). Stretchability of thin metal films on elastomer substrates. Applied Physics Letters.

[13] Xu W, Yang J, Lu T (2011). Ductility of thin copper films on rough polymer substrates. Materials & Design.

[14] Béfahy S, Yunus S, Pardoen T, Bertrand P, Troosters M (2007). Stretchable helical gold conductor on silicone rubber microwire. Applied Physics Letters.

[15] Lambricht N, Pardoen T, Yunus S (2013). Giant stretchability of thin gold films on rough elastomeric substrates. Acta Materialia.

[16] Alford T, Misra E, Bhagat S, Mayer J (2009). Influence of Joule heating during electromigration evaluation of silver lines. Thin Solid Films.

[17] Misra E, Marenco C, Theodore N, Alford T (2005). Failure mechanisms of silver and aluminum on titanium nitride under high current stress. Thin Solid Films.

[18] Pierce D, Brusius P (1997). Electromigration: A review. Microelectronics Reliability.

[19] Li C, Jiang S, Zhang K (2012). Pulse current-assisted hot-forming of light metal alloy. The International Journal of Advanced Manufacturing Technology.

[20] Jones J, Mears L (2013). Thermal response modeling of sheet metals in uniaxial tension during electrically-assisted forming. Journal of Manufacturing Science and Engineering.

[21] Kim M (2014). Electric current-induced annealing during uniaxial tension of aluminum alloy. Scripta Materialia.

[22] Conrad H (2000). Electroplasticity in metals and ceramics. Materials Science and Engineering: A.

[23] Salandro W (2010). Formability of Al 5xxx sheet metals using pulsed current for various heat treatments. Journal of Manufacturing Science and Engineering.

[24] Putz B, Glushko O, Cordill M (2015). Electromigration in gold films on flexible polyimide substrates as a self-healing mechanism. Materials Research Letters.

[25] Laughton, M. & Warne, D. *Electrical engineer’s reference book*. (Newnes, 2003).

[26] Rice J (1976). The Localization of plastic deformation. Theoretical and Applied Mechanics.

[27] Lu N, Wang X, Suo Z, Vlassak J (2009). Failure by simultaneous grain growth, strain localization, and interface debonding in metal films on polymer substrates. Journal of Materials Research.

[28] Lu N, Suo Z, Vlassak J (2010). The effect of film thickness on the failure strain of polymer-supported metal films. Acta Materialia.

[29] Sim G (2011). Improving the stretchability of as-deposited Ag coatings on poly-ethylene-terephthalate substrates through use of an acrylic primer. Journal of Applied Physics.

[30] Yue Y, Zhang J, Wang X (2011). Micro/nanoscale spatial resolution temperature probing for the interfacial thermal characterization of epitaxial graphene on 4H-SiC. Small.

[31] Baris Dogruoz M (2016). Assessment of Joule heating and temperature distribution on printed circuit boards via electrothermal simulations. Journal of Electronic Packaging.

[32] Kim, K. *et al*. Two-barrier stability that allows low-power operation in current-induced domain-wall motion. *Nature Communications* 4, (2013). 10.1038/ncomms3011 23771026

[33] Ko, T., Shellaiah, M. & Sun, K. Thermal and thermoelectric transport in highly resistive single Sb2Se3 nanowires and nanowire bundles. *Scientific Reports* 6, (2016). 10.1038/srep35086 PMC5054389 27713527

[34] Lacy F (2011). Developing a theoretical relationship between electrical resistivity, temperature, and film thickness for conductors. Nanoscale Research Letters.

[35] Ding G, Clavero C, Schweigert D, Le M (2015). Thickness and microstructure effects in the optical and electrical properties of silver thin films. AIP Advances.

[36] Jung Y, Choi Y, Lee H, Lee D (2003). Effects of thermal treatment on the electrical and optical properties of silver-based indium tin oxide/metal/indium tin oxide structures. Thin Solid Films.

[37] Talukdar M, Baker E (1969). Conductivity studies on silver oxide. Solid State Communications.

[38] Freund, L. & Suresh, S. *Thin film materials*. (Cambridge Univ. Press, 2009).

[39] Hau-Riege C (2004). An introduction to Cu electromigration. Microelectronics Reliability.

[40] Li T, Suo Z (2006). Deformability of thin metal films on elastomer substrates. International Journal of Solids and Structures.

[41] Vegt, A. *Polymeren*. (Delft University Press, 1999).

[42] Lu N, Wang X, Suo Z, Vlassak J (2007). Metal films on polymer substrates stretched beyond 50%. Applied Physics Letters.

[43] Tsay, C., Lacour, S., Wagner, S., Li, T. & Suo, Z. How stretchable can we make thin metal films?. in *MRS Proceedings* Vol. 875, pp. O5-5 (Cambridge University Press., 2005).

[44] Hsu Y (2013). Novel strain relief design for multilayer thin film stretchable interconnects. IEEE Transactions on Electron Devices.

[45] Hsu Y (2010). The effect of pitch on deformation behavior and the stretching-induced failure of a polymer-encapsulated stretchable circuit. Journal of Micromechanics and Microengineering.

[46] Taylor R, Boyce C, Boyce M, Pruitt B (2013). Planar patterned stretchable electrode arrays based on flexible printed circuits. Journal of Micromechanics and Microengineering.

[47] Zhang Y (2013). Mechanics of ultra-stretchable self-similar serpentine interconnects. Acta Materialia.

[48] Zhang Y (2013). Experimental and theoretical studies of serpentine microstructures bonded to prestrained elastomers for stretchable electronics. Advanced Functional Materials.

[49] Hsu Y (2011). The effects of encapsulation on deformation behavior and failure mechanisms of stretchable interconnects. Thin Solid Films.

[50] Jablonski M, Bossuyt F, Vanfleteren J, Vervust T, de Vries H (2013). Reliability of a stretchable interconnect utilizing terminated, in-plane meandered copper conductor. Microelectronics Reliability.

[51] Gonzalez M (2011). Design and implementation of flexible and stretchable systems. Microelectronics Reliability.

[52] Lv C, Yu H, Jiang H (2014). Archimedean spiral design for extremely stretchable interconnects. Extreme Mechanics Letters.

[53] Rogers J, Someya T, Huang Y (2010). Materials and mechanics for stretchable electronics. Science.

[54] Kim E (2013). A robust polymer microcable structure for flexible devices. Applied Physics Letters.

[55] Khang DA (2006). Stretchable form of single-crystal silicon for high-performance electronics on rubber substrates. Science.

[56] Feng C (2015). Shrinkage induced stretchable micro-wrinkled reduced graphene oxide composite with recoverable conductivity. Carbon.

[57] Jang S (2016). A three-dimensional metal grid mesh as a practical alternative to ITO. Nanoscale.

[58] Kwon J (2017). Flexible substrates: flexible and transparent Cu electronics by low-temperature acid-assisted laser processing of Cu. Advanced Materials Technologies.

[59] Suh, Y. *et al*. Network patterning: maskless fabrication of highly robust, flexible transparent Cu conductor by random crack network assisted Cu nanoparticle patterning and laser sintering. *Advanced Electronic Materials* 2, (2016).

[60] Hong S (2013). Nonvacuum, maskless fabrication of a flexible metal grid transparent conductor by low-temperature selective laser sintering of nanoparticle ink. ACS Nano.

[61] Yeo J (2012). Next generation non-vacuum, maskless, low temperature nanoparticle ink laser digital direct metal patterning for a large area flexible electronics. PLoS ONE.

[62] Xu F, Zhu Y (2012). Highly conductive and stretchable silver nanowire conductors. Advanced Materials.

[63] Amjadi M, Pichitpajongkit A, Lee S, Ryu S, Park I (2014). Highly stretchable and sensitive strain sensor based on silver nanowire–elastomer nanocomposite. ACS Nano.

[64] Moon H (2017). Ag/Au/Polypyrrole core-shell nanowire network for transparent, stretchable and flexible supercapacitor in wearable energy devices. Scientific Reports.

[65] Hong S (2015). Highly stretchable and transparent metal nanowire heater for wearable electronics applications. Advanced Materials.

[66] Jeong C (2015). A hyper-stretchable elastic-composite energy harvester. Advanced Materials.

[67] Kim K (2015). Highly sensitive and stretchable multidimensional strain sensor with prestrained anisotropic metal nanowire percolation networks. Nano Letters.

[68] Han S (2014). Fast plasmonic laser nanowelding for a Cu-nanowire percolation network for flexible transparent conductors and stretchable electronics. Advanced Materials.

