# Supplementary material for: Self-Heating-Induced Deterioration of Electromechanical Performance in Polymer-Supported Metal Films for Flexible Electronics
Source: Sci Rep. 2017 Oct 2;7:12506. doi: 10.1038/s41598-017-12705-9 (PMC5624894; doi:10.1038/s41598-017-12705-9)
Supplement: Supplementary file 1 — Supplementary Information [file 41598_2017_12705_MOESM1_ESM.pdf]

Supplementary Information

Self-Heating-Induced Deterioration of Electromechanical Performance in Polymer-Supported Metal Films for Flexible Electronics

Dong-Won Jang [1], Jeong-Hwan Lee [2], Ansoon Kim [2, 3], Soon-Bok Lee [4, *], Seong-Gu Hong [2, 3 *]

[1] Reliability Assessment Center, Korea Institute of Machinery and Materials, Daejeon, 34103, Republic of Korea.

[2] Division of Industrial Metrology, Korea Research Institute of Standards and Science, Daejeon, 34113, Republic of Korea.

[3] Department of Nano Science, University of Science and Technology, Daejeon, 34113, Republic of Korea.

[4] Department of Mechanical Engineering, Korea Advanced Institute of Science and Technology, Daejeon, 34141, Republic of Korea.

Corresponding Authors:

* E-mail: sblee@kaist.ac.kr, Soon-Bok Lee

* E-mail: sghong@kriss.re.kr, Seong-Gu Hong

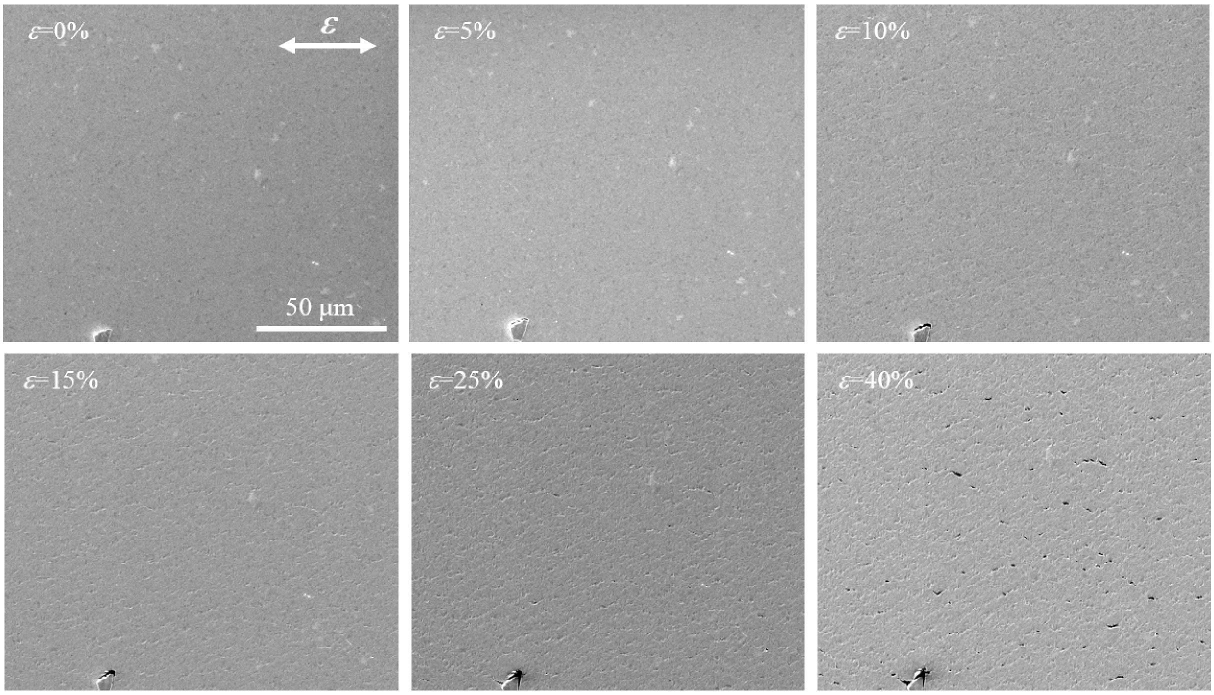

Figure S1. SEM micrographs showing the surface morphology of 400 nm Ag/188 μm PET without an electrical current at six strain levels; all observations were made on the identical location of the sample.

(a)

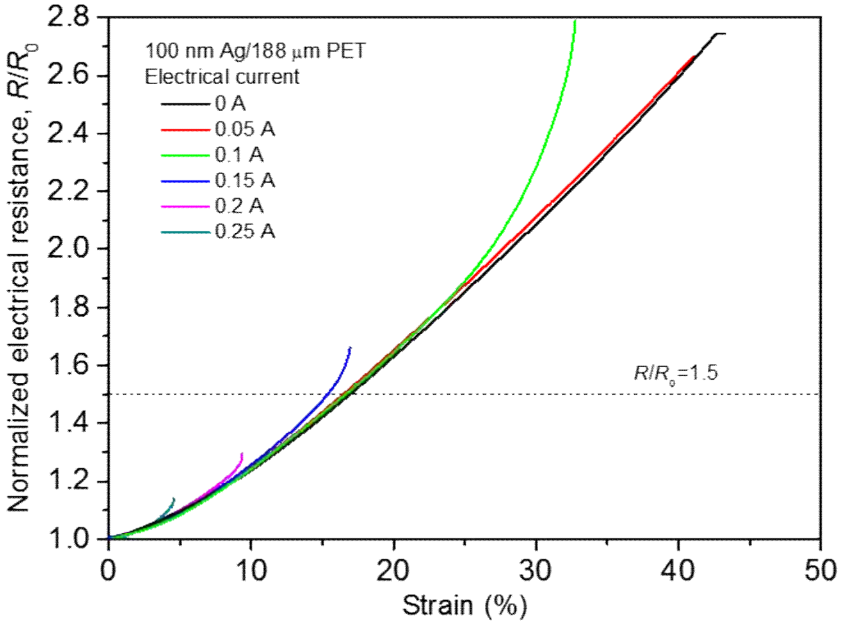

(b)

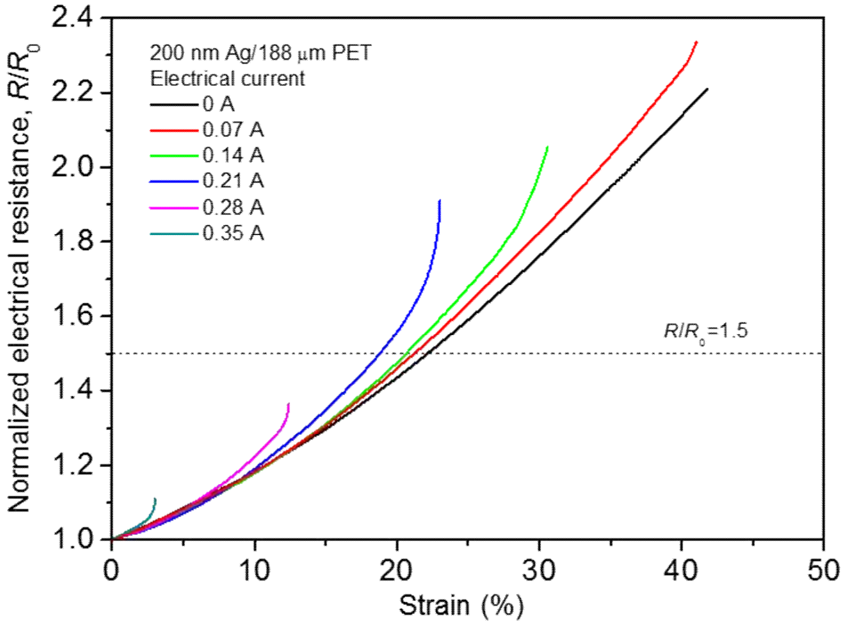

Figure S2. Normalized electrical resistance–strain curve and its variation under an electrical current. (a) 100 nm Ag/188 μm PET. (b) 200 nm Ag/188 μm PET.

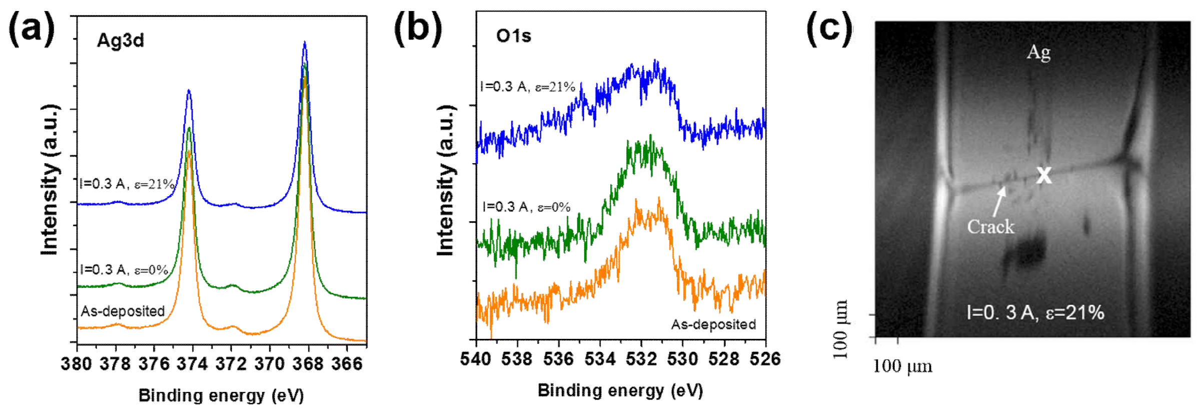

Figure S3. Surface chemical composition analysis of 400 nm Ag/188 μm PET samples subjected to three different test conditions using a scanning X-ray photoelectron spectroscopy (XPS). XPS core level spectra of (a) Ag3d and (b) O1s. (c) A scanning X-ray image showing the XPS measurement location (indicated as "x") of the sample subjected to the test condition of I=0.3 A and ε=21%.

To exmaine the oxidation characteristics of Ag film during the test, the XPS measurements were carried out with a PHI5000 Versa Probe II (Ulvac-PHI) using a monochromatic Al Kα source. The base pressure was below 3×10^{-10} Torr. The instrument work function was calibrated to give the binding energy of 83.96 eV for the Au 4f7/2 core level from a metallic gold film. The X-ray beam diameter was set to 100 μm. The analyzer pass energy was set to 23.5 eV. The binding energy for all spectra was calibrated by referring the C 1s peak from ubiquitous hydrocarbon contamination to 284.8 eV. The peak component fitting (CasaXPS software) was performed by using a Shirley background subtraction and mixed Gaussian-Lorentzian. The fitting parameters were set to reduce the residual standard deviation of the measured and fitted XPS spectra. Three 400 nm Ag/188 μm PET samples were prepared and subjected to three different test conditions, respectively; (1) without an electrical current and mechanical strain (as-deposited – colored orange in Figure S3), (2) with an electrical current of 0.3 A for 0.5 h in the unstrained condition (I=0.3 A, ε=0% – colored green in Figure S3), and (3) with an electrical current of 0.3 A and mechanical strain of 21% (I=0.3 A, ε=21% – colored blue in Figure S3).

Figure S3a and b shows the XPS spectra of Ag3d and O1s core level, respectively, of the samples subjected to three different test condtions; a scanning X-ray image showing the XPS measurement location of the sample subjected to the test condition of I=0.3 A and ε=21% is presented in Figure S3c. The quantitative analysis from the XPS spectra is given in Table S1. The results revealed that the Ag film surfaces of all three samples are composed of mostly metallic Ag with very low oxygen concentrations. The oxygen concentration of the Ag

film surface was 6.3% for the as-deposited film and it slightly increased to 6.6% with the application of an electrical current of 0.3 A in the unstrained condition (i.e., ε=0%). Even with the combined conditions of an electrical current of 0.3 A and mechanical strain of 21% (as shown in Figure S3c, a width-through crack was formed in this sample, indicating that it almost failed electrically), it was still very low (8.6%). Considering the oxygen concentration of a natively oxidized Ag foil is 43%[1], these low oxygen concentrations of < 10% in the three samples indicate that the oxidation of Ag film during the test is not severe. In the XPS measurements, the information depth was about 5 nm (an angle of 45° with respect to the detector). Considering that the XPS measurement is a surface sensitive technique, and thus the composition of the Ag film surface mostly contributes to the measured quantification value, if the Ag film surface is fully oxidized, for example, to 1 nm depth from the surface, the oxygen quantity should be higher than 20%. As shown in Table S1, however, the oxygen concentrations of the three samples were less than 10%, indicating that the oxidation of the Ag films was minor. On the basis of these results, therefore we can conclude that the oxidation effect in Ag film can be negligible in the present study.

Table S1. Surface atomic fractions of Ag and O in 400 nm Ag/188 μm PET samples subjected to three different test conditions.

Sample	Ag	O
As-deposited	93.7%	6.3%
I=0.3 A (0.5 h), ε=0%	93.4%	6.6%
I=0.3 A , ε=21%	91.4%	8.6%

[1] B. V. Crist, *Handbooks of Monochromatic XPS Spectra – The Elements and Native Oxides, Volume 1,* XPS Inter-national LLC: Mountain View, CA, USA, 1999.

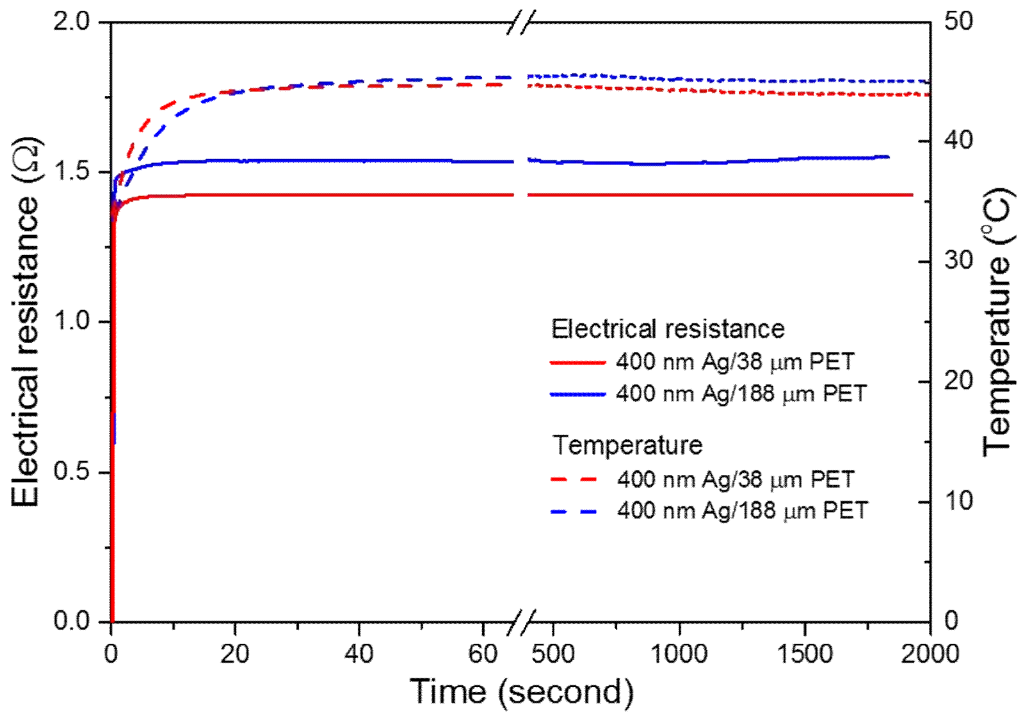

Figure S4. The variation in electrical resistance and temperature of 400 nm Ag/38 and 188 μm PET samples with time under an electrical current of 0.3 A in the unstrained condition.

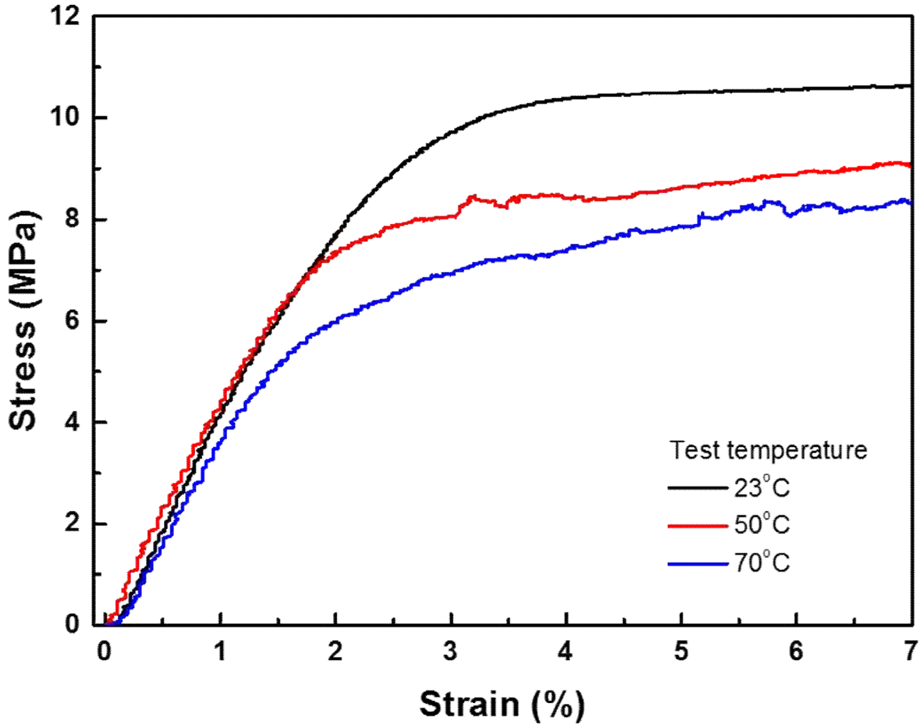

Figure S5. The variation in the tensile curve of 12-μm-thick PET with temperature; the tests were conducted inside a thermal chamber.

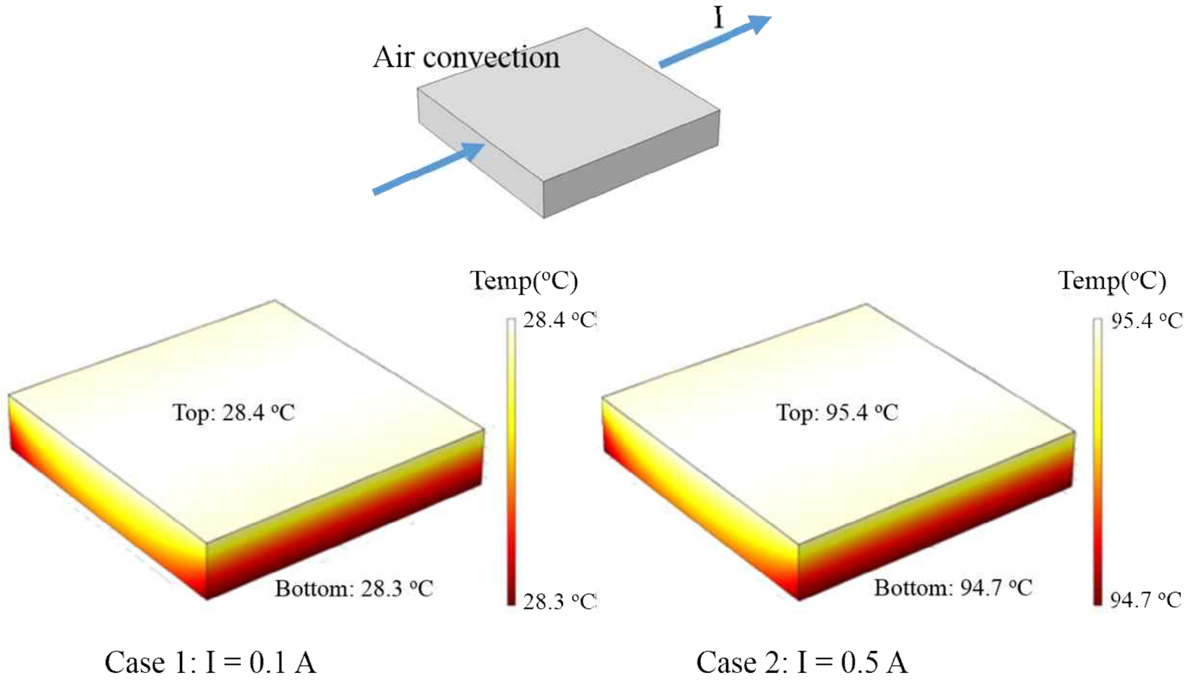

Figure S6. Finite element analysis on the temperature distribution in the 400 nm Ag/188 μm PET sample under electrical currents of 0.1 and 0.5 A in the unstrained condition.

The finite element analysis (FEA) was conducted with a commercial software, Abaqus (Simulia, UK). In the analysis, the FEA model was a 400 nm Ag/188 μm PET with a length of 1 mm and a width of 1 mm. A hexahedral 8–node linear coupled thermal–electrical brick element was used and the convection heat transfer from the sample to the air was assumed. The thermal and electrical properties of the Ag film and PET substrate were quoted from previous studies (**Table S2**).[1–4]

Table S2. The thermal and electrical properties of the Ag film and PET substrate used in the FEA.

Property	Ag	PET
Density (g/cm^3)	10.492	1.38
Thermal conductivity (W/m·K)	429	0.2
Specific heat (kJ/kg·K)	232.87	0.4393

Electrical conductivity (S/m)	6.30×10^7	–
Resistivity (μΩ·m)	0.0147	–
Convective heat transfer (W/m^2K)	10	10

[1] J. E. Mark, *Physical Properties of Polymer Handbook*, Springer, 2007.

[2] R. A. Serway, *Principles of Physics*, Saunders College Pub. 1998.

[3] D. Griffiths, A. Reeves, *Introduction to Electrodynamics*, Prentice Hall, 1999.

[4] D. R. Smith, F. R. Fickett, *J. Res. Natl. Inst. Stand. Technol.* 1995, 100, 119.

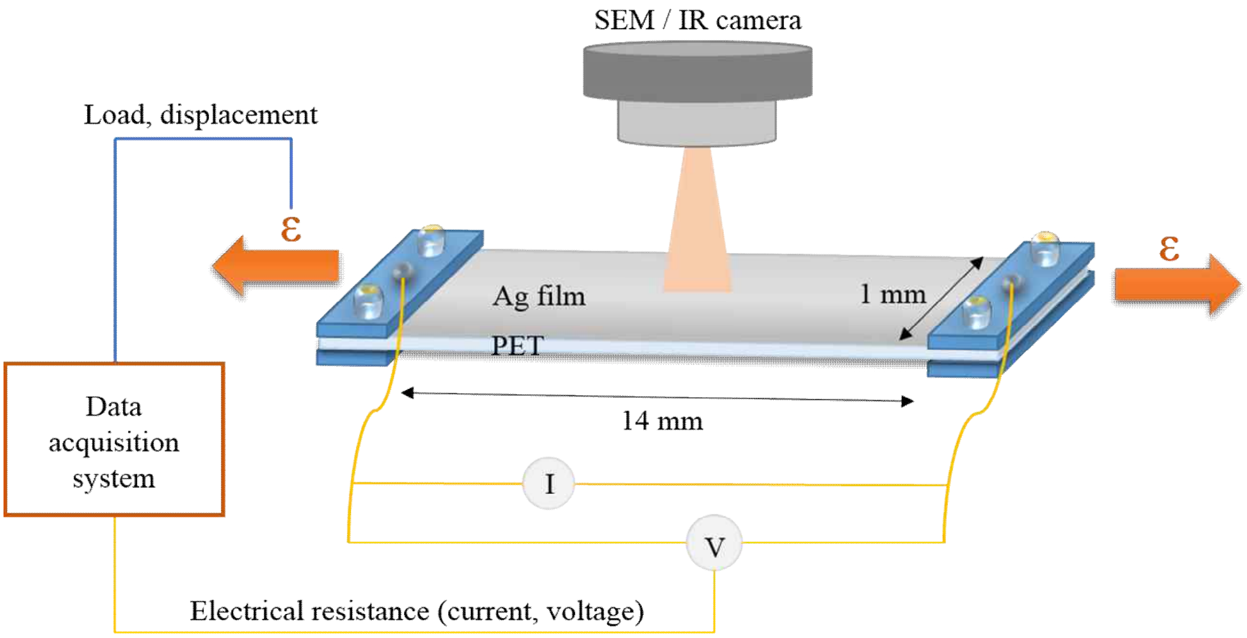

Figure S7. Schematic of the electromechanical tensile testing system integrated with a SEM or an IR camera.
